# Short-term efficacy and safety of 5MHz fractional microneedle radiofrequency for facial rejuvenation: a prospective study

**DOI:** 10.1080/07853890.2026.2708398

**Published:** 2026-07-29

**Authors:** Houhuang Qiu, Xixin Wu, Yutong Wu, Jialu Xu, Yunkai Zhu, Jingchun Chen, Xi Chen, Ping Zhong, Fangfang Wang, Qiusheng Lin, Fei Li, Tianhua Xu

**Affiliations:** Medical Cosmetic Center, Shenzhen Nanshan People’s Hospital, Shenzhen, Guangdong, China

**Keywords:** Facial rejuvenation, fractional microneedle radiofrequency, 5MHz, wrinkles, skin elasticity

## Abstract

**Background:**

Facial aging is a multifactorial process driven by both intrinsic senescence and extrinsic environmental factors. Although fractional microneedle radiofrequency (FMR) is widely used for facial rejuvenation, clinical evidence on 5 MHz FMR remains limited. This prospective study evaluated the efficacy and safety of 5 MHz FMR.

**Methods:**

Twenty participants with facial aging underwent a single full-face 5 MHz FMR treatment. Assessments were performed at baseline and on days 1, 4, 7, 14, and 28 using the MC750 and VISIA systems. Primary outcomes included skin physiological parameters, VISIA absolute parameters, and physician global assessment (PGA). Secondary outcomes included adverse events and patient satisfaction.

**Results:**

Skin hydration showed a transient early decline followed by gradual recovery, whereas melanin remained stable and hemoglobin exhibited a brief early increase before returning toward baseline. Skin elasticity improved significantly at V2 and remained elevated at V5–V6. VISIA analysis demonstrated transient early increases in most parameters, whereas wrinkle scores showed sustained improvement during follow-up. Greater baseline wrinkle severity was associated with greater wrinkle improvement, while subgroup analyses showed no significant differences across Fitzpatrick skin types or age groups. PGA scores progressively improved throughout follow-up. Treatment was well tolerated, with only transient erythema and burning sensations. No serious adverse events, including post-inflammatory hyperpigmentation, were observed, and 85% of participants reported being satisfied or very satisfied.

**Conclusions:**

This prospective study provides preliminary evidence that 5 MHz FMR may offer safe and effective short-term improvement in facial aging-related features, particularly wrinkles and skin elasticity. The treatment was associated with rapid recovery, limited irritation, and a low incidence of pigment-related adverse events.

## 1. Introduction

Against the backdrop of accelerating global population aging, demand for appearance management has continued to rise, making facial rejuvenation an increasingly important focus in aesthetic medicine [1,2]. Notably, age-related facial changes may emerge as early as the third decade of life [3]. Facial aging is now understood as a multifactorial process driven by both intrinsic senescence and extrinsic environmental insults. At the tissue level, it is characterized by loss of collagen and elastin, along with fragmentation of collagen fibers. Clinically, these changes manifest as wrinkles, pigmentary irregularities, skin laxity, and reduced elasticity [4–6]. Energy-based modalities are now widely used in facial rejuvenation [7–9]. Among them, fractional microneedle radiofrequency (FMR) combines controlled mechanical injury with radiofrequency-induced thermal stimulation to create focal dermal microinjury, thereby promoting collagen remodeling and elastic fiber regeneration. It has therefore emerged as a promising rejuvenation strategy [10–12]. In current practice, most FMR devices operate at 1 to 2 MHz [13–16]. Evidence on 5 MHz FMR remains limited. Because radiofrequency penetration depth is inversely related to frequency [17], a 5 MHz setting may generate more confined radiofrequency thermal zones under comparable energy delivery, potentially reducing excessive thermal spread while maintaining clinical efficacy, and possibly lowering the risk of post-inflammatory hyperpigmentation (PIH) and related adverse events [18,19]. To date, clinical data on 5 MHz FMR for facial rejuvenation are still lacking. This prospective study was therefore designed to systematically evaluate its efficacy and safety, with the aim of providing preliminary clinical evidence for its use in facial rejuvenation.

## 2. Materials and methods

This prospective study was conducted in accordance with the ethical principles of the Declaration of Helsinki and was approved by the Ethics Committee of Shenzhen Nanshan People’s Hospital (approval No. ky-2025-110302). The study protocol was also registered in the Chinese Medical Research Registration and Filing Information System (registration No. MR-44-26-017340). Only de-identified clinical data were used to protect patient privacy, and written informed consent was obtained from all participants before treatment.

### 2.1. Patient selection criteria

A total of 20 participants aged 35 to 55 years were enrolled between December 24, 2025, and February 25, 2026. All had Fitzpatrick skin types II to IV and a clinical diagnosis of facial aging, mainly characterized by facial wrinkles, enlarged pores, and skin laxity. Exclusion criteria were as follows: facial inflammation, infection, ulceration, or other dermatologic conditions such as vitiligo, psoriasis, or eczema; a history of keloid formation; any facial rejuvenation treatment within the previous 3 months, including laser, radiofrequency, ultrasound, chemical peeling, or botulinum toxin injection; marked sun exposure or tanning within 1 month before enrollment; systemic disease, immunodeficiency, current immunosuppressive therapy, or psychiatric disorders; and pregnancy or lactation. All participants were fully informed of the study objectives, procedures, potential benefits, and possible risks, and provided written informed consent before treatment. In addition, all participants provided separate written informed consent for the use and publication of their clinical data and facial photographs, including identifiable facial features, for research and academic purposes.

### 2.2. Devices and materials

The following devices and materials were used: a 5 MHz fractional microneedle radiofrequency device (XEMIS Medical Technology Co., Ltd., Guangdong, China), VISIA imaging system (Canfield Scientific, Inc., New Jersey, USA), Multi Skin Test Center MC 750 (Courage + Khazaka Electronic GmbH, Cologne, Germany) and 5% compound lidocaine cream (Tongfang Pharmaceutical Group Co., Ltd., Beijing, China).

### 2.3. Treatment protocol

**Figure 1.**
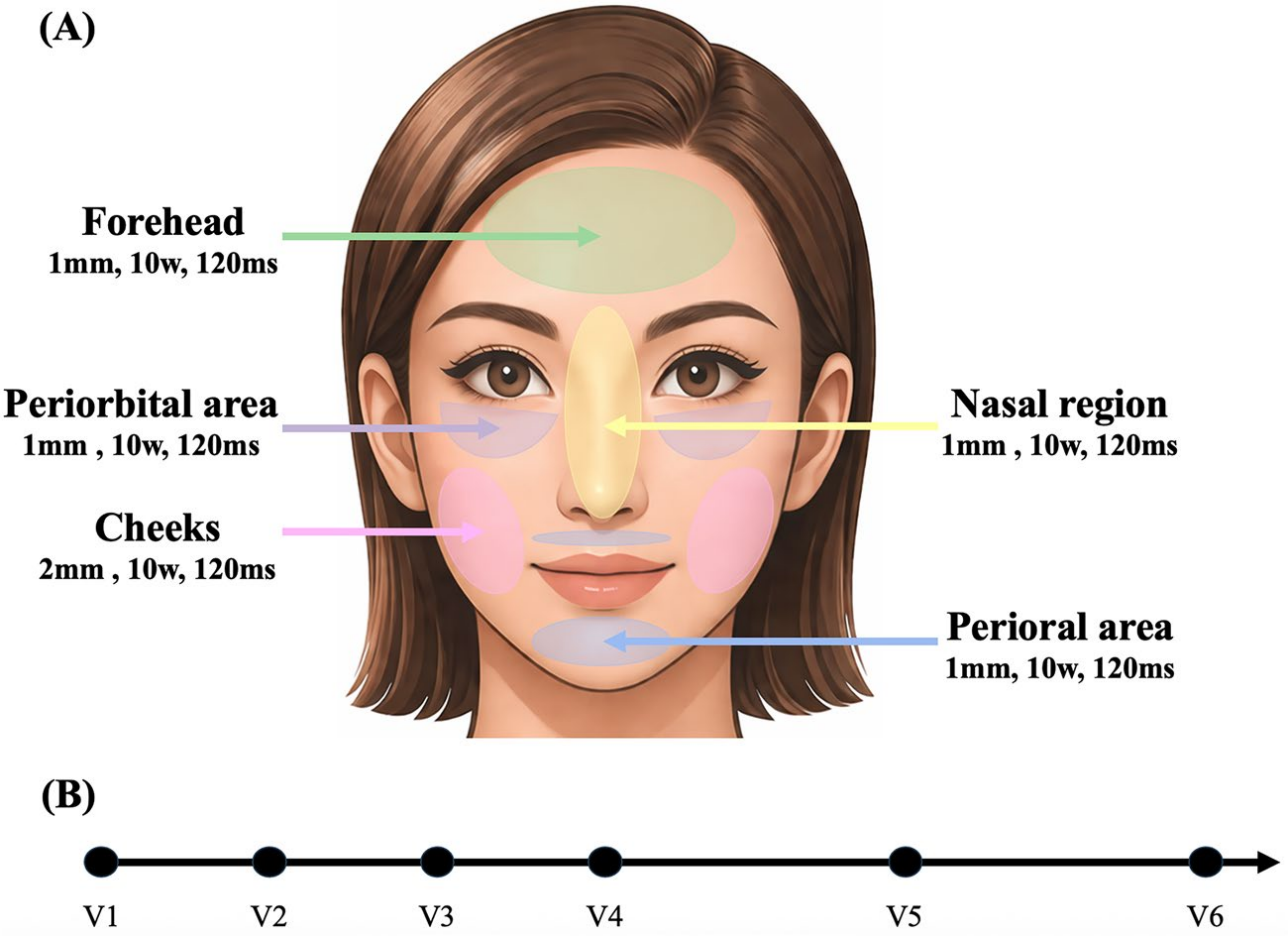
Schematic illustration of treatment parameters and follow-up schedule. (A) Full-face treatment areas and parameter settings for 5 MHz FMR. The cheeks were treated at a depth of 2 mm, with a power of 10 W and a pulse duration of 120 ms; all remaining facial areas were treated at a depth of 1 mm, with a power of 10 W and a pulse duration of 120 ms. (B) Follow-up schedule. Assessments were performed at baseline before treatment (V1) and on days 1 (V2), 4 (V3), 7 (V4), 14 (V5), and 28 (V6) after treatment.

All participants received full-face FMR treatment. Before treatment, 5% lidocaine cream was evenly applied to the face for 1 h to achieve topical anesthesia, followed by routine disinfection with 75% medical alcohol. Treatment parameters were as follows: for the cheeks, a needle depth of 2 mm, power of 10 W, and pulse duration of 120 ms; for the remaining facial areas, a needle depth of 1 mm, power of 10 W, and pulse duration of 120 ms. A non-insulated microneedle was used, and a total of 830 treatment pulses were delivered in a single session (Figure 1A). Routine skincare was resumed 24 h later. Participants were advised to maintain adequate skin hydration and moisturization during recovery, and strict use of a broad-spectrum sunscreen (SPF 50) was recommended after re-epithelialization. No additional aesthetic procedures or energy-based treatments were allowed during the follow-up period.

### 2.4. Treatment evaluation

At baseline (V1) and on days 1, 4, 7, 14, and 28 after treatment (V2–V6), facial assessments were performed using the MC 750 and VISIA systems (Figure 1B). All VISIA images were acquired using the same device under standardized conditions, including consistent lighting, head positioning, facial expression, and camera settings, according to the manufacturer’s recommended imaging protocol. Primary outcome measures included skin physiological parameters obtained with the MC 750, namely Skin Hydration, Melanin, Hemoglobin, and Skin Elasticity, together with VISIA absolute parameters, including Spots, Wrinkles, Texture, Pores, UV Spots, Brown Spots, Red Areas, and Porphyrins. These findings were further integrated with the Physician Global Assessment (PGA) for overall evaluation [20]. During follow-up from V2 to V6, PGA scoring was completed by one aesthetic physician who was not involved in treatment, based on comparison of VISIA images at each follow-up visit with baseline images. The scoring system was defined as follows: 1 = complete resolution, 2 = marked improvement, 3 = moderate improvement, 4 = mild improvement, 5 = no improvement, and 6 = worsened. Secondary outcomes included adverse events and patient satisfaction, which were collected throughout treatment and follow-up *via* online questionnaires or outpatient visits.

### 2.5. Statistical analysis

Statistical analyses were performed using SPSS software (version 27.0; IBM Corp., Armonk, NY, USA). Data normality was first assessed with the Kolmogorov–Smirnov test. Normally distributed continuous variables are presented as mean ± standard deviation (SD) and were compared using appropriate parametric tests. Non-normally distributed continuous variables are expressed as M (P_25_, P_75_) and were analyzed with the Wilcoxon rank-sum test. Categorical variables were compared using the chi-square test or Fisher’s exact test, as appropriate. Ordinal data were analyzed with the Wilcoxon rank-sum test. Linear regression was used to examine the association between baseline scores and the degree of improvement, with the coefficient of determination (R^2^) used to estimate the strength of correlation. A *p* < 0.05 was considered statistically significant. To control for inflation of type I error arising from multiple comparisons, p values obtained from pairwise comparisons were adjusted using the Holm–Bonferroni procedure. A two-sided adjusted *p*  <0.05 was considered statistically significant.

## 3. Results

### 3.1. Baseline characteristics of the study population

**Figure 2.**
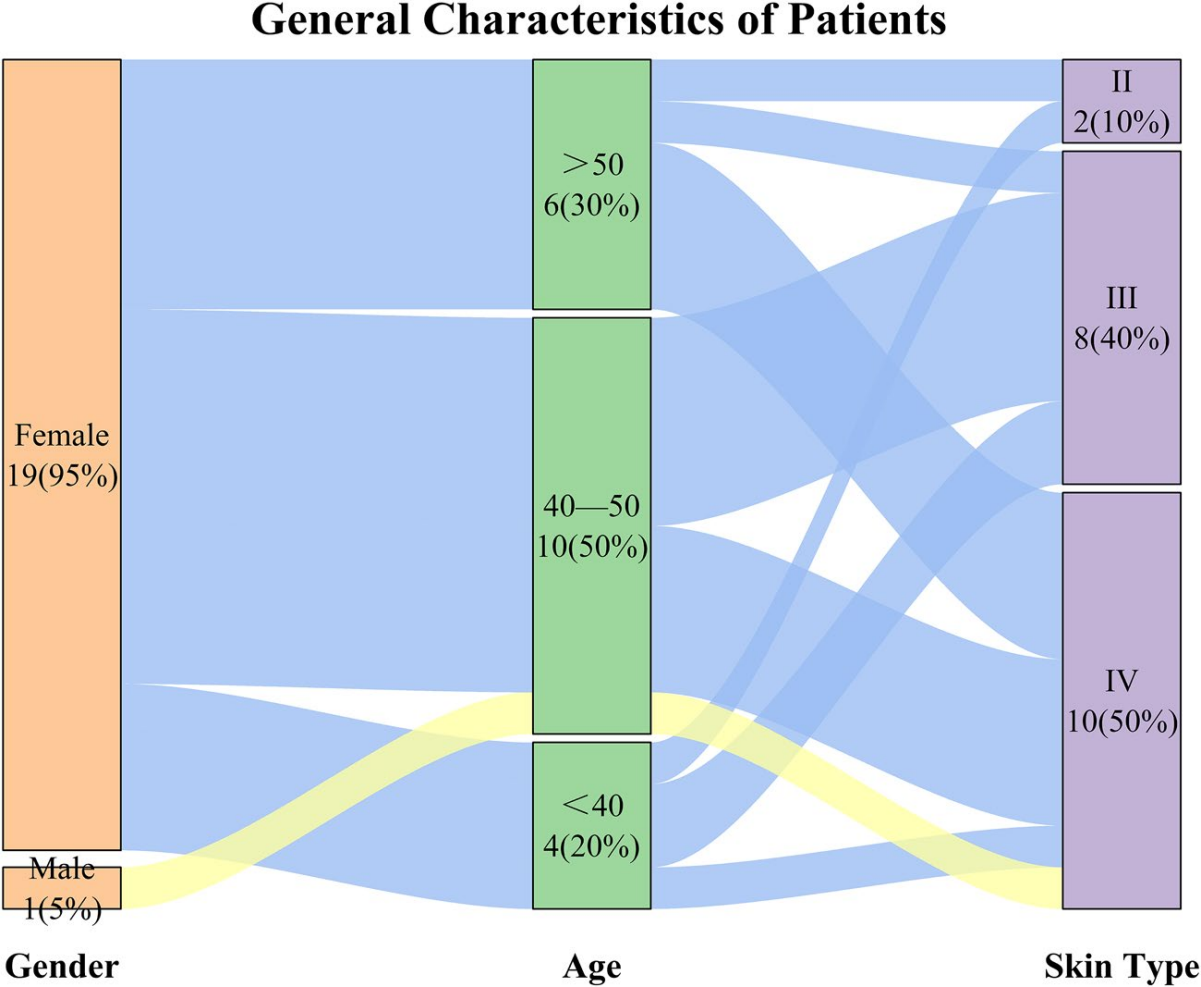
Distribution of baseline participant characteristics. A total of 20 participants were included, comprising 19 women (95%) and 1 man (5%). The largest age subgroup was 40–50 years (10, 50%), followed by >50 years (6, 30%) and <40 years (4, 20%). Fitzpatrick skin type IV was the most common (10, 50%), followed by type III (8, 40%) and type II (2, 10%).

A total of 20 participants were included in this study, comprising 19 women (95%) and 1 man (5%). The largest age subgroup was 40–50 years (10, 50%), followed by those older than 50 years (6, 30%) and those younger than 40 years (4, 20%). Fitzpatrick skin type IV was the most common (10, 50%), followed by type III (8, 40%) and type II (2, 10%). Across age strata, most participants had Fitzpatrick type III or IV skin, with type IV predominating in the 40–50-year group, indicating that the study population mainly consisted of middle-aged individuals with relatively darker skin types (Figure 2).

### 3.2. Assessment of primary outcomes

**Figure 3.**
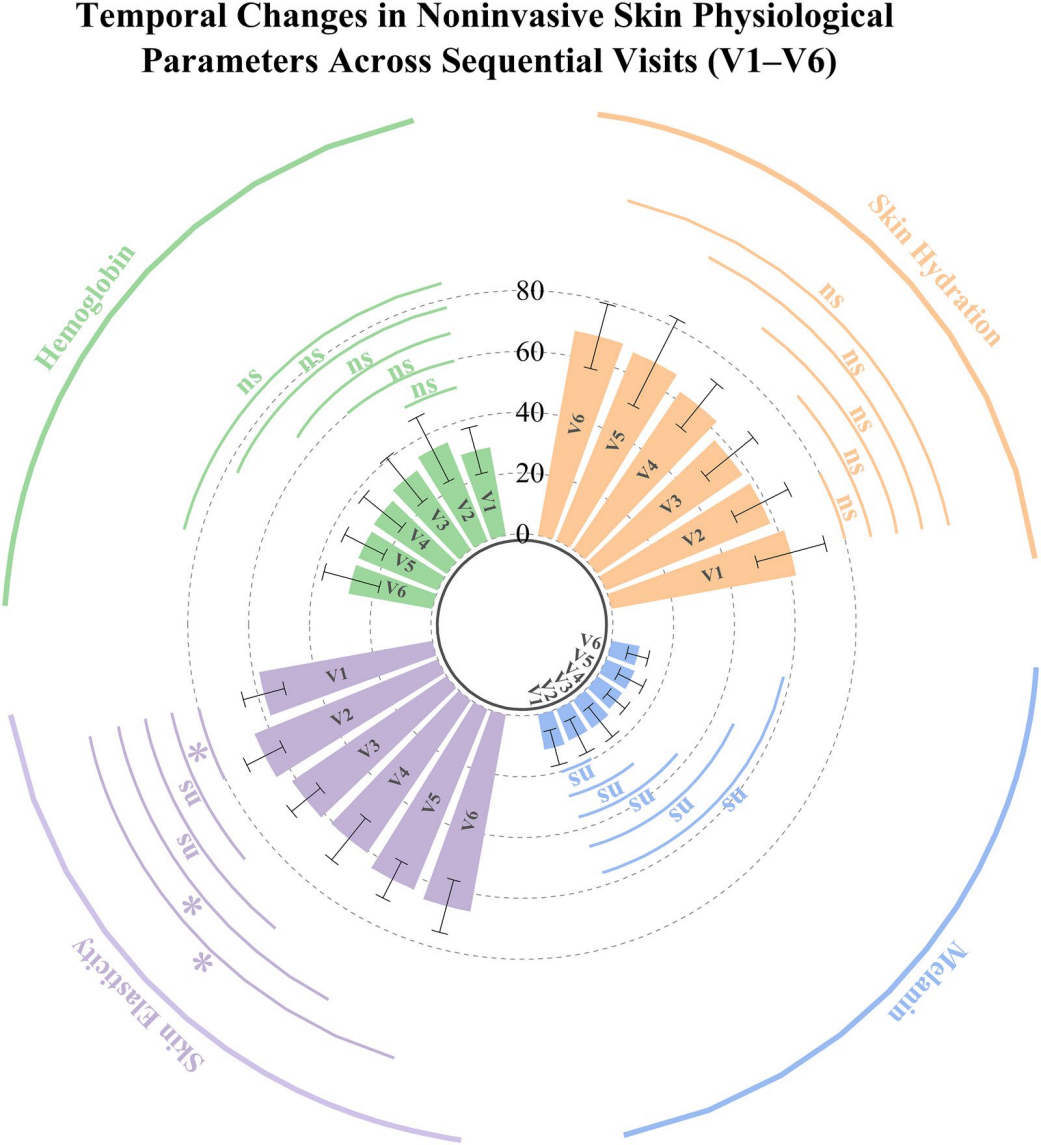
Changes in skin physiological parameters at baseline and follow-up visits. Skin hydration, melanin, hemoglobin, and skin elasticity were measured with the MC 750 at baseline (V1) and at each follow-up visit (V2–V6). The figure shows temporal changes in each parameter together with the corresponding statistical comparisons. “ns” indicates no significant difference; * adjusted *p* < 0.05, ** adjusted *p* < 0.01, and *** adjusted *p* < 0.001.

**Figure 4.**
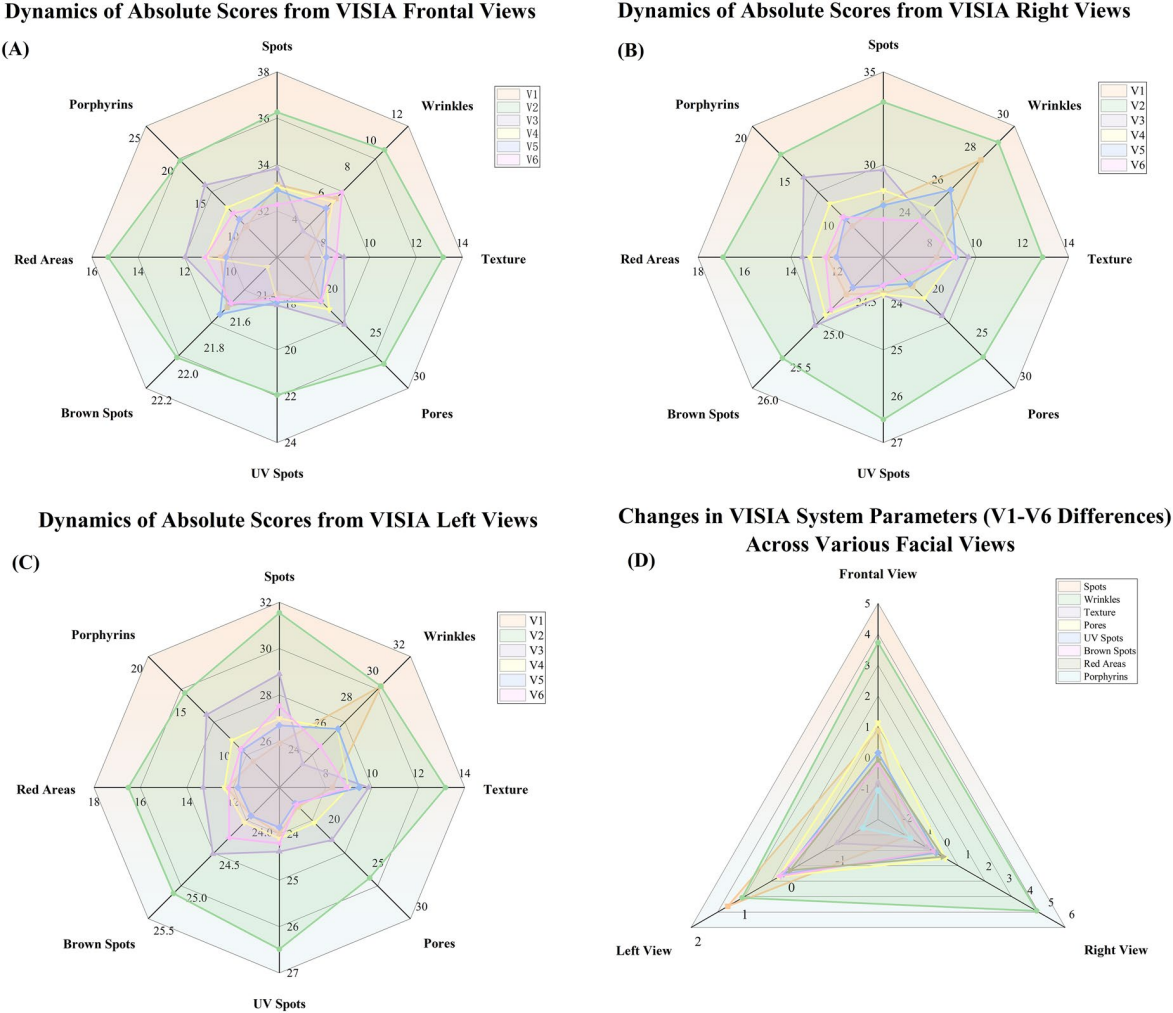
Temporal profiles of VISIA absolute parameters across different views. (A–C) Dynamic changes in VISIA absolute parameters, including spots, wrinkles, texture, pores, UV spots, brown spots, red areas, and porphyrins, in the frontal, right lateral, and left lateral views at baseline (V1) and follow-up visits (V2–V6). (D) Analysis of the V1–V6 differences in VISIA parameters across views, showing the overall magnitude of change for each parameter during follow-up.

**Figure 5.**
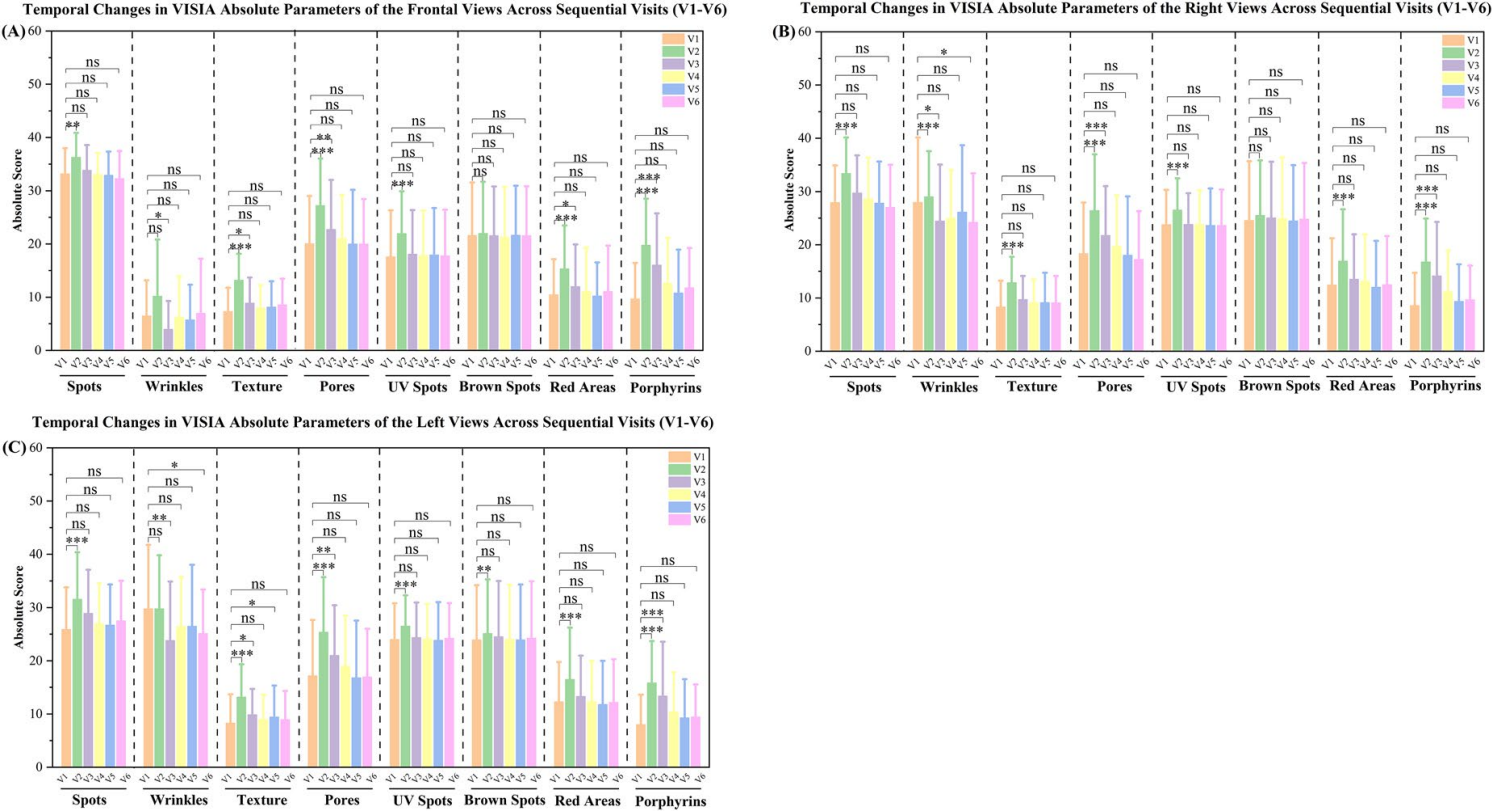
Dynamic changes in VISIA absolute parameters across different views and follow-up visits. (A–C) VISIA absolute parameters, including spots, wrinkles, texture, pores, UV spots, brown spots, red areas, and porphyrins, in the frontal, right lateral, and left lateral views at baseline (V1) and follow-up visits (V2–V6). “ns” indicates no significant difference; * adjusted *p*<0.05, ** adjusted *p*<0.01, and *** adjusted *p*<0.001.

Skin physiological parameters exhibited distinct temporal changes during follow-up (Figure 3, Table S1). Skin hydration showed a transient early decline followed by gradual recovery, whereas melanin and hemoglobin remained relatively stable, with no significant differences from baseline after Holm–Bonferroni correction (all adjusted *p *> 0.05). In contrast, skin elasticity increased significantly at V2 and remained significantly elevated at V5 and V6 (all adjusted *p* < 0.05). VISIA absolute parameters showed distinct but generally transient changes across the frontal, right lateral, and left lateral views (Figure 4A–C). Most parameters exhibited an early increase at V2, followed by gradual recovery toward baseline during subsequent follow-up. Spots, UV spots, and red areas showed significant but transient increases at V2 in all three views, whereas porphyrin scores remained significantly elevated through V3 before returning to baseline thereafter (all adjusted *p*  <0.05). Texture and pore scores also increased significantly during the early post-treatment period, although the duration of significance varied among facial views. In contrast, wrinkle scores showed consistent improvement, with significant reductions observed at V3 in all three views and sustained improvement through V6 in both lateral views (all adjusted *p*  <0.05). Brown spots remained largely unchanged throughout follow-up, except for a transient increase at V2 in the left lateral view (adjusted *p*  <0.05) (Figure 5 and Table S2). Analysis of V1–V6 change values further demonstrated that wrinkles exhibited the greatest overall improvement across all facial views, particularly in the right lateral view, whereas changes in spots, pores, red areas, brown spots, and porphyrins were comparatively modest (Figure 4D).

**Figure 6.**
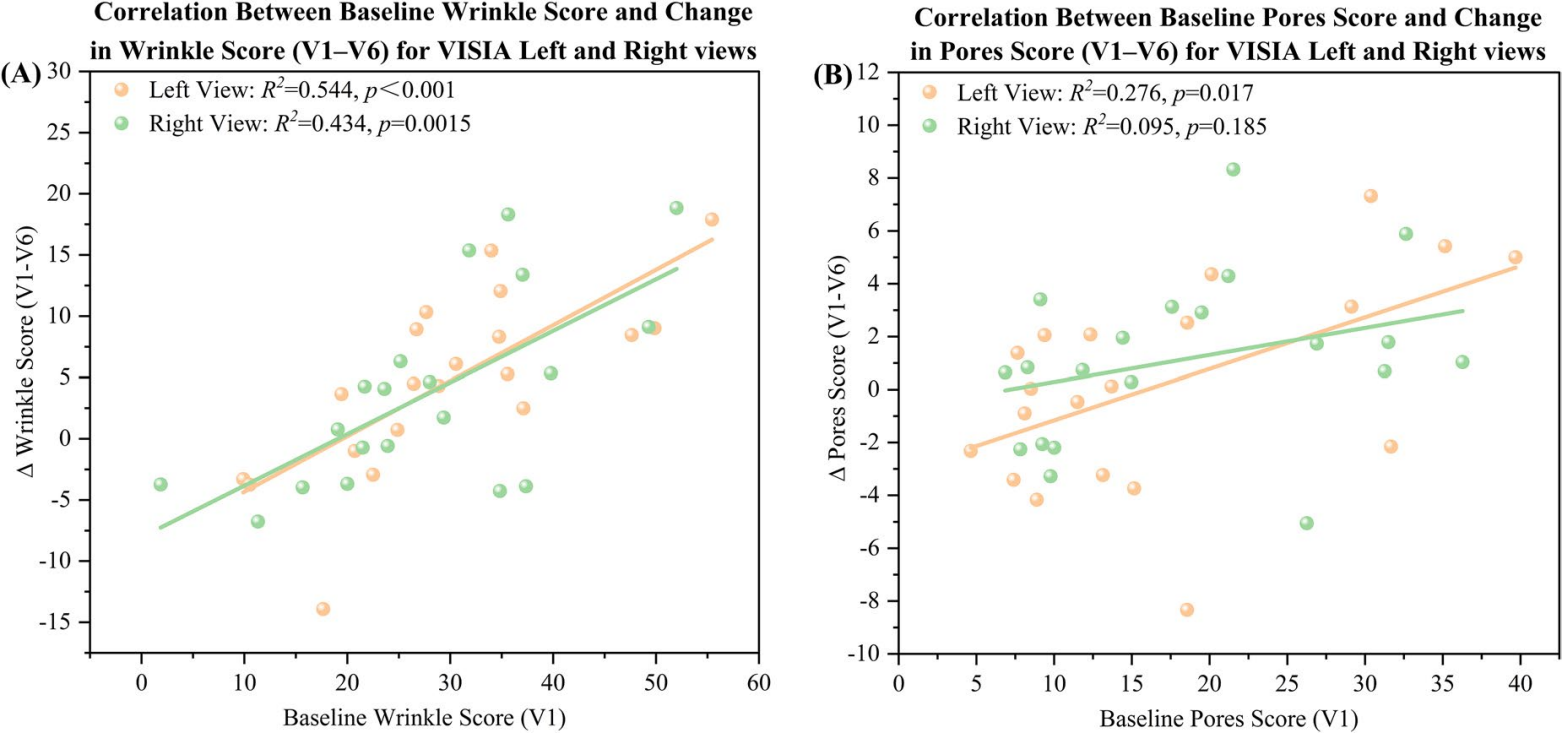
Correlation between baseline wrinkle and pore scores and treatment-related improvement. (A) Linear regression analysis of baseline wrinkle scores and wrinkle improvement, defined as the V1–V6 change in wrinkle scores. (B) Linear regression analysis of baseline pore scores and pore improvement, defined as the V1–V6 change in pore scores. Fitted results are shown separately for the left and right lateral views.

Linear regression analysis demonstrated that higher baseline wrinkle scores were significantly associated with greater wrinkle improvement in both the right and left lateral views (R^2^  =0.434, *p* = 0.0015; R^2^  =0.544, *p*  <0.001) (Figure 6A). Baseline pore scores were also positively associated with pore improvement in the left lateral view (R^2^  =0.276, *p*  =0.017), whereas no significant association was observed in the right lateral view (*p*>0.05) (Figure 6B). Subgroup analysis showed no significant differences in the V1–V6 changes in skin elasticity or total wrinkle scores, defined as the sum of wrinkle scores across all views, across Fitzpatrick skin types or age groups. For skin elasticity, the improvement tended to be greater in Fitzpatrick type III skin and in participants younger than 40 years, and smaller in type IV skin and those older than 50 years, although the between-group differences were not significant (*p*  =0.289, *p*  =0.172). For total wrinkle scores, greater reductions were observed in type II skin and in the 40–50-year group, again without significant differences across skin type or age strata (*p*  =0.750, *p*  =0.842) (Tables 1 and 2).

**Table 1.** Subgroup analysis based on skin elasticity scores [(mean ± SD), M (P_25_, P_75_)].

Subgroup	Number (n)	V1	V6	Difference (V1-V6)	*p*
Fitzpatrick skin type					
Type II	2	58.150 ± 0.707	66.925 ± 9.511	8.775 ± 10.218	0.289
Type III	8	55.319 ± 7.956	67.981 ± 6.904	12.663 ± 10.270	
Type IV	10	60.060 ± 6.622	63.905 ± 10.596	3.845 ± 12.036	
Age (years)					
40 <	4	56.200(53.475,61.850)	70.100(64.263,79.688)	14.750(9.938,17.838)	0.172
40–50	10	56.410 ± 6.425	65.840 ± 9.104	9.430 ± 9.303	
> 50	6	61.108 ± 9.173	62.158 ± 8.547	1.050 ± 15.499	

**Table 2.** Subgroup analysis based on total wrinkle scores [(mean ± SD), M (P_25_, P_75_)].

Subgroup	Number (n)	V1	V6	Difference (V1-V6)	*p*
Fitzpatrick skin type					
Type II	2	63.517 ± 9.019	50.636 ± 5.559	12.881 ± 3.461	0.750
Type III	8	60.005 ± 19.109	53.847 ± 14.636	6.157 ± 17.648	
Type IV	10	67.642 ± 35.777	59.387 ± 26.323	8.255 ± 15.611	
Age (years)					
40 <	4	64.948(50.307,79.413)	52.084(46.122,71.185)	8.131(3.050,14.096)	0.842
40–50	10	66.502 ± 23.503	57.284 ± 16.412	9.218 ± 19.365	
> 50	6	52.790(35.485,77.474)	54.539 ± 31.395	5.281 ± 13.622	

**Figure 7.**
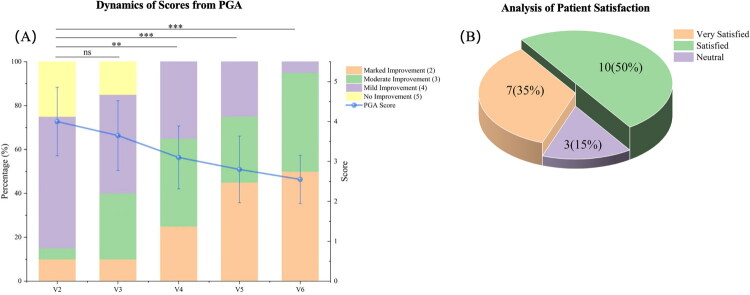
Dynamic changes in PGA scores and patient satisfaction. (A) Mean Physician Global Assessment (PGA) scores and the distribution of response categories at follow-up visits V2–V6. The stacked bars show the proportions of marked improvement, moderate improvement, mild improvement, and no improvement, while the line indicates the mean PGA score. (B) Patient satisfaction outcomes. Of the 20 participants, 10 (50%) were satisfied, 7 (35%) were very satisfied, and 3 (15%) reported neutral satisfaction. *p  <0.05, **p  <0.01, and ***p  <0.001.

PGA scores demonstrated progressive overall improvement during follow-up. Compared with V2, PGA scores were significantly lower at V4–V6 (all *p*  <0.01), whereas no significant difference was observed between V2 and V3 (*p*  >0.05). Consistent with this trend, the distribution of PGA categories shifted from predominantly mild or no improvement at the early follow-up visits to mainly moderate and marked improvement by V6 (Figure 7A).

### 3.3. Assessment of secondary outcomes

After treatment, all participants developed varying degrees of erythema, and 13 (65%) experienced noticeable burning or stinging sensations. No acute complications, such as burns, were observed immediately after the procedure. Participants were advised to intensify post-treatment hydration and moisturization. These reactions were generally mild, transient, and reversible. Erythema lasted approximately 1–4 days, whereas burning or stinging resolved spontaneously within 2–4 h. No serious adverse events, including post-inflammatory hyperpigmentation, scarring, wound infection, acneiform eruption, or folliculitis, were observed during follow-up.

Patient satisfaction was favorable, with 17 of 20 participants (85%) reporting satisfaction or high satisfaction. The remaining three participants (15%) rated the treatment outcome as neutral, and no participant reported dissatisfaction, indicating a high level of overall treatment acceptance (Figure 7B).

## 4. Discussion

The goal of facial rejuvenation extends beyond transient improvement in surface appearance and, more importantly, involves long-term optimization of overall skin quality through dermal remodeling, extracellular matrix renewal, and restoration of barrier function [21]. One of the key strengths of FMR lies in its ability to deliver both mechanical and thermal stimulation. By creating controlled focal injury within the dermis, it initiates a subsequent repair and remodeling response, thereby promoting neocollagenesis, extracellular matrix turnover, and improved biomechanical properties of the skin [22,23]. In routine clinical practice, most FMR systems operate at 1–2 MHz. By contrast, the 5 MHz setting used in the present study may theoretically allow more focused energy delivery and a more confined thermal profile, potentially maintaining rejuvenation efficacy while minimizing thermal spread and excessive stimulation of adjacent tissue [24]. This potential balance between precision and safety provided an important rationale for the present evaluation of 5 MHz FMR.

**Figure 8.**
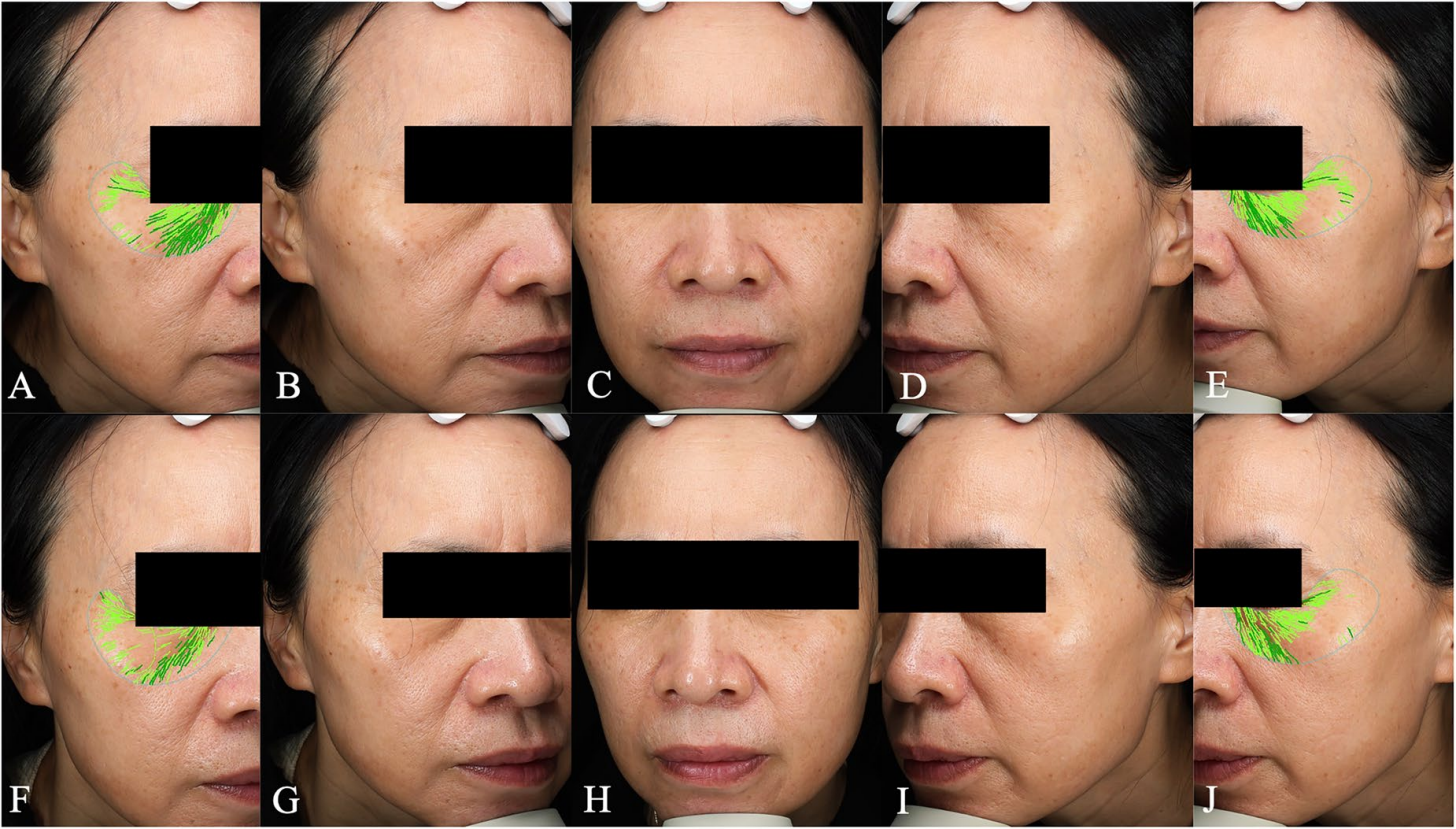
Representative VISIA images of a 55-year-old female participant. (A–E) Baseline (V1) VISIA images showing, in order, right-sided wrinkle analysis, right lateral view, frontal view, left lateral view, and left-sided wrinkle analysis; (F–J) Corresponding VISIA images at V6, showing, in order, right-sided wrinkle analysis, right lateral view, frontal view, left lateral view, and left-sided wrinkle analysis.

**Figure 9.**
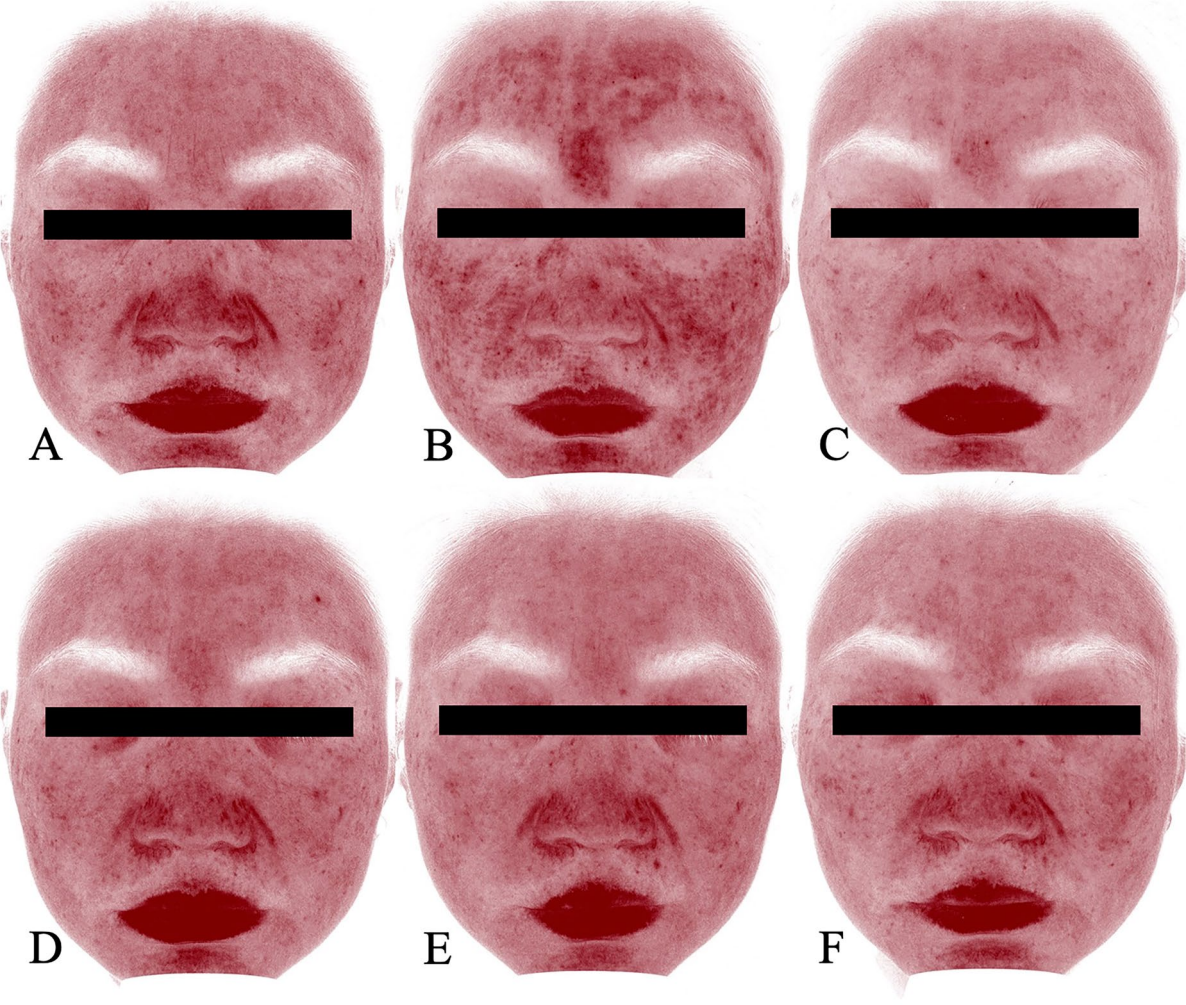
VISIA red area images of a 42-year-old female participant. (A–F) VISIA red area images obtained at V1 through V6 across the follow-up period.

Our findings showed that after 5 MHz FMR treatment, both skin physiological parameters and VISIA absolute values underwent dynamic changes, suggesting that this modality can induce a continuous short-term process that evolves from an early treatment response to subsequent repair. Skin hydration showed a mild decline in the early post-treatment phase, followed by a gradual recovery over subsequent follow-up visits. This pattern may reflect transient impairment of barrier integrity after microneedle penetration and thermal exposure [25]. Increased transepidermal water loss immediately after treatment may have contributed to the temporary reduction in measured hydration levels. As the stratum corneum barrier progressively recovered and the acute inflammatory response subsided, skin hydration gradually returned toward baseline [26]. In parallel, hemoglobin levels showed a slight increase at V2–V3 and then gradually returned to near-baseline values, suggesting a brief phase of local vasodilation and inflammatory activation that did not progress to sustained irritation. Melanin levels fluctuated only minimally and showed no significant increase overall, indirectly supporting favorable pigmentary safety of 5 MHz FMR in individuals with relatively darker skin types. Notably, skin elasticity improved significantly as early as V2, then entered a short plateau phase before rising again at V5–V6. This pattern suggests that the effect on skin biomechanical properties may not follow a simple linear course. The early improvement may be related to tissue contraction and immediate dermal tightening, whereas the later increase more likely reflects the progressive contribution of neocollagenesis and matrix remodeling [27,28]. VISIA findings further provided objective imaging support for this pattern of early response followed by later remodeling. Most parameters showed a transient increase at V2 and then gradually declined, suggesting that microneedle injury and radiofrequency heating may temporarily aggravate surface roughness, erythema, and certain texture-related features in the early post-treatment phase. This should not be interpreted as true worsening of skin aging, but more likely reflects short-lived inflammation, edema, and barrier disturbance. Among all parameters, wrinkles showed the most pronounced improvement, and significant differences from baseline remained evident in both lateral views at V6, suggesting a relatively stable anti-wrinkle effect of 5 MHz FMR. This wrinkle reduction is likely related to FMR-induced dermal collagen remodeling and elastic fiber regeneration [29], which is also consistent with the later increase in skin elasticity observed in this study (Figure 8). By contrast, the net reductions in UV spots, brown spots, and red areas were more limited, although their temporal patterns still appeared clinically meaningful. Red areas rose briefly in the early phase and then rapidly returned to near-baseline levels. This trend paralleled the quick normalization of hemoglobin and may indicate that the local inflammatory response induced by 5 MHz FMR was relatively mild and short-lived (Figure 9). Previous studies have reported erythema and edema lasting from several days to weeks after FMR treatment [30,31]. In the present study, both red area and hemoglobin values returned to baseline relatively quickly, which may suggest that 5 MHz FMR preserves clinical efficacy while offering a potential advantage in reducing skin irritation and shortening the duration of inflammatory responses. Brown spots and UV spots, meanwhile, did not show a sustained increase after treatment and gradually declined at later time points. Together with the absence of post-inflammatory hyperpigmentation during follow-up, this finding suggests that the more confined thermal profile of 5 MHz FMR may have avoided marked pigment activation, thereby potentially lowering the risk of pigment-related adverse events. The pattern of improvement also varied slightly by view. Differences in the magnitude of change between the frontal and lateral views may reflect regional variation in skin thickness, sebum distribution, facial muscle activity, and optical acquisition angle [32–35].

Linear regression analysis further suggested that greater baseline aging severity was generally associated with larger post-treatment improvement, indicating that 5 MHz FMR may elicit a stronger treatment response in individuals with more pronounced wrinkles and pores. This observation is broadly consistent with clinical experience, as more evident dermal laxity, collagen loss, and textural irregularity may provide greater scope for energy-induced remodeling, making objective improvement easier to detect [36,37]. The absence of a significant association for pores in the right lateral view may reflect the limited sample size, baseline asymmetry between facial sides, or local variability in VISIA measurements. Subgroup analysis further showed no significant differences in improvements in skin elasticity or total wrinkle scores across Fitzpatrick skin types or age strata, suggesting that this treatment may be applicable across different skin types and age groups. PGA findings also supported the objective results. Mean PGA scores declined steadily from V2 to V6, and the response profile shifted from predominantly mild or no improvement at earlier visits to mainly moderate and marked improvement at later visits, indicating that the rejuvenating effect of 5 MHz FMR was not immediate but emerged progressively during repair and remodeling. But PGA assessments were performed by a single independent evaluator, and no formal inter-rater reliability analysis was conducted. Therefore, observer bias cannot be completely excluded, despite the use of objective instrumental measurements as the primary outcome measures. Patient satisfaction was likewise high, with 85% reporting satisfaction or high satisfaction. Taken together, these findings suggest that 5 MHz FMR not only yields favorable short-term objective outcomes but is also well accepted by patients, particularly those seeking improvement in wrinkles and overall skin quality while remaining concerned about downtime and adverse effects.

In terms of safety, no serious adverse events were observed in this study. Erythema and transient discomfort were most likely related to the local inflammatory response induced by microneedle penetration and radiofrequency heating and were considered expected short-term reactions after FMR treatment. Of particular interest, most participants had Fitzpatrick skin types III–IV, yet no post-inflammatory hyperpigmentation was observed during follow-up, a finding of particular clinical relevance in Asian populations [38]. Given the theoretical profile of 5 MHz FMR, the higher frequency may allow a shallower and more confined pattern of thermal deposition, potentially reducing excessive heat spread while still providing adequate dermal stimulation. This may offer an advantage in limiting pigment-related adverse events. That said, this hypothesis still requires confirmation in larger cohorts and in direct comparative studies with conventional 1–2 MHz systems.

The follow-up period was set at 4 weeks primarily to minimize potential confounding factors, including seasonal environmental influences and interindividual differences in home skincare practices [39]. Although the follow-up duration was relatively short, clear clinical and imaging improvements were still observed within this limited window, providing preliminary support for the efficacy and safety of 5 MHz FMR in facial rejuvenation. This study nonetheless has several limitations. It was a single-center prospective observational study with a small sample size, and no direct comparison was made with conventional 1–2 MHz FMR or other energy-based devices. In addition, only a single treatment session was evaluated, and the study population was limited to Fitzpatrick skin types II–IV. Therefore, the cumulative effects of repeated treatment sessions and the generalizability of the findings to a broader range of skin types remain unclear. As such, the relative advantages of the 5 MHz setting in terms of treatment efficacy and complication control remain to be clarified. Further large-scale randomized controlled studies with more diverse patient populations and longer follow-up periods are warranted to better define the therapeutic profile and clinical value of 5 MHz FMR in facial rejuvenation.

## 5. Conclusion

This prospective study provides preliminary evidence that 5 MHz FMR may offer safe and effective short-term improvement in facial aging-related features, particularly wrinkles and skin elasticity. The treatment was associated with rapid recovery, limited irritation, and a low incidence of pigment-related adverse events.

## Supplementary Material

Supplementary Material.docx

## Data Availability

Data are available on reasonable requests from the corresponding author.
