# Supplementary material for: Short-term efficacy and safety of 5MHz fractional microneedle radiofrequency for facial rejuvenation: a prospective study
Source: Ann Med. 2026 Jul 29;58(1):2708398. doi: 10.1080/07853890.2026.2708398 (PMC13421117; doi:10.1080/07853890.2026.2708398)
Supplement: Supplementary Material.docx [file IANN_A_2708398_SM1661.docx]

| **Table S1. Comparison of Noninvasive Skin Physiological Parameters Between Baseline (V1) and Follow-up Visits (V2–V6)** | | | |
| --- | --- | --- | --- |
| Parameter | Comparison | p | Adjusted p |
| Skin Hydration | V1 vs V2 | 0.262 | 0.726 |
|  | V1 vs V3 | 0.172 | 0.688 |
|  | V1 vs V4 | 0.785 | 0.785 |
|  | V1 vs V5 | 0.242 | 0.726 |
|  | V1 vs V6 | 0.037 | 0.185 |
| Hemoglobin | V1 vs V2 | 0.064 | 0.320 |
|  | V1 vs V3 | 0.471 | 1.000 |
|  | V1 vs V4 | 0.841 | 1.000 |
|  | V1 vs V5 | 0.550 | 1.000 |
|  | V1 vs V6 | 0.609 | 1.000 |
| Skin Elasticity | V1 vs V2 | 0.002 | 0.010 |
|  | V1 vs V3 | 0.148 | 0.296 |
|  | V1 vs V4 | 0.331 | 0.331 |
|  | V1 vs V5 | 0.007 | 0.024 |
|  | V1 vs V6 | 0.006 | 0.024 |
| Melanin | V1 vs V2 | 0.575 | 1.000 |
|  | V1 vs V3 | 0.775 | 1.000 |
|  | V1 vs V4 | 0.248 | 0.992 |
|  | V1 vs V5 | 0.370 | 1.000 |
|  | V1 vs V6 | 0.146 | 0.730 |

| **Table S2. Comparison of VISIA Absolute Parameters Between Baseline (V1) and Follow-up Visits (V2–V6)** | | | | |
| --- | --- | --- | --- | --- |
| Parameter | View | Comparison | p | Adjusted p |
| Spots | Frontal | V1 vs V2 | 0.001 | 0.005 |
|  |  | V1 vs V3 | 0.390 | 1.000 |
|  |  | V1 vs V4 | 0.890 | 1.000 |
|  |  | V1 vs V5 | 0.693 | 1.000 |
|  |  | V1 vs V6 | 0.207 | 0.828 |
|  | Right | V1 vs V2 | $<$0.001 | $<$0.001 |
|  |  | V1 vs V3 | 0.114 | 0.456 |
|  |  | V1 vs V4 | 0.351 | 0.837 |
|  |  | V1 vs V5 | 0.737 | 0.837 |
|  |  | V1 vs V6 | 0.279 | 0.837 |
|  | Left | V1 vs V2 | $<$0.001 | $<$0.001 |
|  |  | V1 vs V3 | 0.025 | 0.100 |
|  |  | V1 vs V4 | 0.601 | 0.702 |
|  |  | V1 vs V5 | 0.351 | 0.702 |
|  |  | V1 vs V6 | 0.179 | 0.537 |
| Wrinkles | Frontal | V1 vs V2 | 0.191 | 0.573 |
|  |  | V1 vs V3 | 0.006 | 0.030 |
|  |  | V1 vs V4 | 0.681 | 0.956 |
|  |  | V1 vs V5 | 0.040 | 0.160 |
|  |  | V1 vs V6 | 0.478 | 0.956 |
|  | Right | V1 vs V2 | $<$0.001 | $<$0.001 |
|  |  | V1 vs V3 | 0.006 | 0.024 |
|  |  | V1 vs V4 | 0.112 | 0.224 |
|  |  | V1 vs V5 | 0.302 | 0.302 |
|  |  | V1 vs V6 | 0.012 | 0.036 |
|  | Left | V1 vs V2 | 0.990 | 0.990 |
|  |  | V1 vs V3 | 0.002 | 0.010 |
|  |  | V1 vs V4 | 0.081 | 0.207 |
|  |  | V1 vs V5 | 0.069 | 0.207 |
|  |  | V1 vs V6 | 0.011 | 0.044 |
| Texture | Frontal | V1 vs V2 | $<$0.001 | $<$0.001 |
|  |  | V1 vs V3 | 0.005 | 0.020 |
|  |  | V1 vs V4 | 0.166 | 0.166 |
|  |  | V1 vs V5 | 0.028 | 0.084 |
|  |  | V1 vs V6 | 0.028 | 0.084 |
|  | Right | V1 vs V2 | $<$0.001 | $<$0.001 |
|  |  | V1 vs V3 | 0.052 | 0.208 |
|  |  | V1 vs V4 | 0.085 | 0.255 |
|  |  | V1 vs V5 | 0.155 | 0.255 |
|  |  | V1 vs V6 | 0.101 | 0.255 |
|  | Left | V1 vs V2 | $<$0.001 | $<$0.001 |
|  |  | V1 vs V3 | 0.006 | 0.024 |
|  |  | V1 vs V4 | 0.122 | 0.178 |
|  |  | V1 vs V5 | 0.010 | 0.030 |
|  |  | V1 vs V6 | 0.089 | 0.178 |
| Pores | Frontal | V1 vs V2 | $<$0.001 | $<$0.001 |
|  |  | V1 vs V3 | 0.002 | 0.008 |
|  |  | V1 vs V4 | 0.306 | 0.918 |
|  |  | V1 vs V5 | 0.977 | 0.977 |
|  |  | V1 vs V6 | 0.969 | 0.977 |
|  | Right | V1 vs V2 | $<$0.001 | $<$0.001 |
|  |  | V1 vs V3 | $<$0.001 | $<$0.001 |
|  |  | V1 vs V4 | 0.176 | 0.435 |
|  |  | V1 vs V5 | 0.911 | 0.911 |
|  |  | V1 vs V6 | 0.145 | 0.435 |
|  | Left | V1 vs V2 | $<$0.001 | $<$0.001 |
|  |  | V1 vs V3 | 0.002 | 0.008 |
|  |  | V1 vs V4 | 0.087 | 0.261 |
|  |  | V1 vs V5 | 0.881 | 1.000 |
|  |  | V1 vs V6 | 0.788 | 1.000 |
| UV Spots | Frontal | V1 vs V2 | $<$0.001 | $<$0.001 |
|  |  | V1 vs V3 | 0.331 | 1.000 |
|  |  | V1 vs V4 | 0.552 | 1.000 |
|  |  | V1 vs V5 | 0.425 | 1.000 |
|  |  | V1 vs V6 | 0.555 | 1.000 |
|  | Right | V1 vs V2 | $<$0.001 | $<$0.001 |
|  |  | V1 vs V3 | 0.921 | 1.000 |
|  |  | V1 vs V4 | 0.927 | 1.000 |
|  |  | V1 vs V5 | 0.634 | 1.000 |
|  |  | V1 vs V6 | 0.654 | 1.000 |
|  | Left | V1 vs V2 | $<$0.001 | $<$0.001 |
|  |  | V1 vs V3 | 0.266 | 1.000 |
|  |  | V1 vs V4 | 0.759 | 1.000 |
|  |  | V1 vs V5 | 0.683 | 1.000 |
|  |  | V1 vs V6 | 0.401 | 1.000 |
| Brown Spots | Frontal | V1 vs V2 | 0.037 | 0.185 |
|  |  | V1 vs V3 | 0.823 | 1.000 |
|  |  | V1 vs V4 | 0.117 | 0.468 |
|  |  | V1 vs V5 | 0.695 | 1.000 |
|  |  | V1 vs V6 | 0.940 | 1.000 |
|  | Right | V1 vs V2 | 0.028 | 0.140 |
|  |  | V1 vs V3 | 0.073 | 0.292 |
|  |  | V1 vs V4 | 0.232 | 0.696 |
|  |  | V1 vs V5 | 0.881 | 0.881 |
|  |  | V1 vs V6 | 0.411 | 0.822 |
|  | Left | V1 vs V2 | 0.002 | 0.010 |
|  |  | V1 vs V3 | 0.073 | 0.292 |
|  |  | V1 vs V4 | 0.601 | 1.000 |
|  |  | V1 vs V5 | 0.911 | 1.000 |
|  |  | V1 vs V6 | 0.218 | 0.654 |
| Red Areas | Frontal | V1 vs V2 | $<$0.001 | $<$0.001 |
|  |  | V1 vs V3 | 0.011 | 0.044 |
|  |  | V1 vs V4 | 0.179 | 0.537 |
|  |  | V1 vs V5 | 0.737 | 0.822 |
|  |  | V1 vs V6 | 0.411 | 0.822 |
|  | Right | V1 vs V2 | $<$0.001 | $<$0.001 |
|  |  | V1 vs V3 | 0.057 | 0.228 |
|  |  | V1 vs V4 | 0.100 | 0.300 |
|  |  | V1 vs V5 | 0.204 | 0.408 |
|  |  | V1 vs V6 | 0.881 | 0.881 |
|  | Left | V1 vs V2 | $<$0.001 | $<$0.001 |
|  |  | V1 vs V3 | 0.052 | 0.208 |
|  |  | V1 vs V4 | 0.911 | 1.000 |
|  |  | V1 vs V5 | 0.313 | 0.939 |
|  |  | V1 vs V6 | 0.627 | 1.000 |
| Porphyrins | Frontal | V1 vs V2 | $<$0.001 | $<$0.001 |
|  |  | V1 vs V3 | $<$0.001 | $<$0.001 |
|  |  | V1 vs V4 | 0.057 | 0.171 |
|  |  | V1 vs V5 | 0.627 | 0.627 |
|  |  | V1 vs V6 | 0.067 | 0.171 |
|  | Right | V1 vs V2 | $<$0.001 | $<$0.001 |
|  |  | V1 vs V3 | $<$0.001 | $<$0.001 |
|  |  | V1 vs V4 | 0.067 | 0.201 |
|  |  | V1 vs V5 | 0.737 | 0.737 |
|  |  | V1 vs V6 | 0.296 | 0.592 |
|  | Left | V1 vs V2 | $<$0.001 | $<$0.001 |
|  |  | V1 vs V3 | $<$0.001 | $<$0.001 |
|  |  | V1 vs V4 | 0.067 | 0.201 |
|  |  | V1 vs V5 | 0.287 | 0.287 |
|  |  | V1 vs V6 | 0.108 | 0.216 |
